# Models predict change in plasma triglyceride concentrations and long-chain n-3 polyunsaturated fatty acid proportions in healthy participants after fish oil intervention

**DOI:** 10.3389/fnut.2022.989716

**Published:** 2022-10-25

**Authors:** Tilly I. T. Potter, Graham W. Horgan, Anne J. Wanders, Elizabeth H. Zandstra, Peter L. Zock, Helena L. Fisk, Anne M. Minihane, Philip C. Calder, John C. Mathers, Baukje de Roos

**Affiliations:** ^1^The Rowett Institute, University of Aberdeen, Aberdeen, United Kingdom; ^2^Biomathematics and Statistics Scotland, Aberdeen, United Kingdom; ^3^Unilever Foods Innovation Centre, Wageningen, Netherlands; ^4^Division of Human Nutrition and Health, Wageningen University and Research, Wageningen, Netherlands; ^5^Faculty of Medicine, University of Southampton, Southampton, United Kingdom; ^6^Norwich Medical School, University of East Anglia, Norwich, United Kingdom; ^7^NIHR Southampton Biomedical Research Centre, University Hospital Southampton NHS Foundation Trust, University of Southampton, Southampton, United Kingdom; ^8^Human Nutrition Research Centre, Population Health Sciences Institute, Newcastle University, Newcastle upon Tyne, United Kingdom

**Keywords:** precision nutrition, omega-3, fish oil, statistical modeling, secondary analysis, crossover study

## Abstract

**Introduction:**

Substantial response heterogeneity is commonly seen in dietary intervention trials. In larger datasets, this variability can be exploited to identify predictors, for example genetic and/or phenotypic baseline characteristics, associated with response in an outcome of interest.

**Objective:**

Using data from a placebo-controlled crossover study (the FINGEN study), supplementing with two doses of long chain n-3 polyunsaturated fatty acids (LC n-3 PUFAs), the primary goal of this analysis was to develop models to predict change in concentrations of plasma triglycerides (TG), and in the plasma phosphatidylcholine (PC) LC n-3 PUFAs eicosapentaenoic acid (EPA) + docosahexaenoic acid (DHA), after fish oil (FO) supplementation. A secondary goal was to establish if clustering of data prior to FO supplementation would lead to identification of groups of participants who responded differentially.

**Methods:**

To generate models for the outcomes of interest, variable selection methods (forward and backward stepwise selection, LASSO and the Boruta algorithm) were applied to identify suitable predictors. The final model was chosen based on the lowest validation set root mean squared error (RMSE) after applying each method across multiple imputed datasets. Unsupervised clustering of data prior to FO supplementation was implemented using k-medoids and hierarchical clustering, with cluster membership compared with changes in plasma TG and plasma PC EPA + DHA.

**Results:**

Models for predicting response showed a greater TG-lowering after 1.8 g/day EPA + DHA with lower pre-intervention levels of plasma insulin, LDL cholesterol, C20:3*n*-6 and saturated fat consumption, but higher pre-intervention levels of plasma TG, and serum IL-10 and VCAM-1. Models also showed greater increases in plasma PC EPA + DHA with age and female sex. There were no statistically significant differences in PC EPA + DHA and TG responses between baseline clusters.

**Conclusion:**

Our models established new predictors of response in TG (plasma insulin, LDL cholesterol, C20:3n-6, saturated fat consumption, TG, IL-10 and VCAM-1) and in PC EPA + DHA (age and sex) upon intervention with fish oil. We demonstrate how application of statistical methods can provide new insights for precision nutrition, by predicting participants who are most likely to respond beneficially to nutritional interventions.

## Introduction

There is often a large degree of variability in physiological outcomes within nutritional intervention studies (1–3). This means that some participants respond beneficially to an intervention, while others may respond poorly or not at all (4). Precision nutrition aims to identify the drivers of these differences, and predict who may respond beneficially (5). While determining response at the level of a single individual requires multiple measurements over time, e.g., through an N-of-1 study (6), predictors of response to outcomes at a group level may be identified through appropriate application of statistical methods in well-powered studies (7). Understanding associations between phenotype, genotype and physiological response could lead to greater understanding of the mechanisms responsible for differential response to interventions, and provide a rational basis for the tailoring of dietary interventions to subgroups of the population (8–10).

Response heterogeneity is seen for physiological markers that can have daily fluctuations, such as plasma triglyceride (TG) concentration (3), as well as those that can vary over longer time periods, such as plasma long-chain n-3 polyunsaturated fatty acids (LC n-3 PUFAs, also called omega-3 fatty acids) (9, 11). Plasma concentration of TG and LC n-3 PUFAs are common outcomes of interest in LC n-3 PUFA supplementation trials. Fish oil (FO) is a good source of LC n-3 PUFAs, including eicosapentaenoic acid (EPA) and docosahexaenoic acid (DHA), which have been shown to lower TG concentrations in many intervention trials (12). An increase in the omega-3 index (EPA + DHA as a percentage of total fatty acids in erythrocyte membranes) has been linked to lower risk of cardiovascular disease (13, 14).

The FINGEN study was a double-blind, placebo-controlled crossover study investigating the effects of low (0.7 g EPA + DHA/d, 0.7FO) and medium (1.8 g EPA + DHA/d, 1.8FO) doses of fish oil for 8 weeks on cardiovascular disease risk biomarkers, including plasma TG concentration (15). The FINGEN study revealed greater body weight-adjusted increases in plasma phosphatidylcholine (PC) DHA in men compared with women, with lowering of TG concentration in response to 1.8FO being 3 times greater in males, and a trend toward reductions seen in apolipoprotein E4 (APOE4) carriers (15). Significantly higher baseline TG concentrations were observed in APOE4 carriers compared with E2 and E3 carriers (16). However, previous analyses only stratified by two factors (sex and APOE genotype) but did not exploit the whole dataset to identify which of the many available variables could best predict response to intervention, in terms of reductions in plasma TG and increases in PC EPA + DHA after supplementation.

Using data from the FINGEN study, the primary goal of this analysis was to identify the predictors that best explain the response heterogeneity of plasma TG and plasma PC EPA + DHA to LC n-3 PUFA supplementation, using variable selection methods and validation approaches. The second goal was to determine whether unsupervised analysis of pre-intervention and baseline data could help to identify groups that responded differentially to LC n-3 PUFA supplementation.

## Methods

### FINGEN study design and participants

Characteristics of the participants recruited to the FINGEN study, and the methods used, have been reported in full elsewhere (15, 16). The original study was approved by the ethics committee at each of the four universities involved in the study (15). Briefly, 312 healthy participants who consumed oily fish less than once a week, recruited at 4 centers in the UK, completed three 8-week intervention periods. They consumed a control oil (an 80:20 blend of palm oil and soybean oil) containing no EPA or DHA, 0.7FO and 1.8FO in a random order, separated by two 12-week washout periods. The participant flow chart can be found in Supplementary Figure 1.

Before and after each intervention period, a fasting (12 h-fast) blood sample was collected for the measurement of plasma lipids, apolipoproteins, glucose and insulin concentrations (15). Plasma was used for assessment of fatty acid proportions (15); PC is the most abundant phospholipid in plasma (17) and plasma PC EPA + DHA has been shown to be a suitable biomarker of LC n-3 PUFA intake in long-term studies (18). Plasma PC fatty acid composition was determined by gas chromatography.

For genotyping, the buffy layer was collected from an ethylenediaminetetraacetic acid (EDTA) tube (BD Biosciences, San Diego, CA, USA) and genomic DNA was extracted using a DNA extraction kit (Qiagen, Hildenberg, Germany), following the manufacturer’s instructions. SNP genotyping was conducting using a commercial SNP genotyping service, TaqMan™ SNP Genotyping Assay, human, Applied Biosystems.

### Data overview

Data were received in Excel sheets and amalgamated to form a single dataset. The dataset included descriptive and physiological variables, dietary intake data, information on single nucleotide polymorphisms (SNPs) and plasma PC fatty acid data. All variables included in this analysis can be found in Supplementary Table 1. Due to lack of variability, SNPs with ≥ 99% genotype similarity between participants were removed. Data from two participants were removed due to > 10% missing data. The complete dataset was imported into R (version 4.1.0), which was used for all statistical analyses. A copy of the (un-imputed) dataset was created, with numeric variables standardized for comparing coefficients in the final models.

Prior to multiple imputation, all SNPs and sex (M/F) were coded as factor variables. SNP data was coded 1–3, with 1 corresponding to two reference alleles and 2 and 3 corresponding to one and two non-reference alleles, respectively. All other numeric variables were mean-centered to improve interpretability of the final model coefficients (19). Using the *mice* package in R (20), collinear variables were removed prior to multiple imputation, which replaced missing values with estimates from the distribution of the remaining data (20). Missing data per variable was between 0 and 6%, with total missing data just under 1%. Multiple imputation generated 5 complete imputed (independent) datasets. 5 imputations were chosen and deemed acceptable due to the size of the dataset and low amount of total missing data, meaning the variation between the imputed datasets was expected to be low (20). To improve statistical power, SNPs were converted back to numeric variables after imputation, aside from codes designating APOE variant (2 = E2/E2 + E2/E3, 3 = E3/E3, 4 = E3/E4 + E4/E4; *rs429358* and *rs7412*) and endothelial nitric oxide synthase (eNOS, *rs1799983*; 1 = GG, 2 = GT, 3 = TT) due to their inclusion as basic characteristics in the original dataset. Details of all SNPs and their reference IDs can be found in Supplementary Table 1.

Each imputed dataset was divided into a dataset containing all baseline variables and data collected prior to the 0.7FO treatment arm (0.7FO dataset), and a dataset containing all baseline variables and data collected prior to the 1.8FO treatment arm (1.8FO dataset), to examine the predictors of response prior to each treatment arm separately. In total, each imputed dataset contained 98 variables (including volunteer identifier and outcome variables) and 310 participants.

This study focused on two outcomes: change in plasma TG concentration, and change in plasma PC EPA + DHA calculated from the difference in EPA + DHA proportion, as a percentage of total fatty acids, pre- and post-fish oil supplementation. For the purpose of this report, these outcomes are referred to as change scores. Outcomes were used on a continuous scale rather than as a dichotomous classification (e.g., response/non-response) to maximize use of information and statistical power (21, 22). To examine if there were significant differences in the outcomes of interest between treatment arms, ANOVA tests with Huynh-Feldt correction were conducted (23). To determine whether supervised analysis for both outcomes was appropriate after each FO intervention, the standard deviations (SDs) of the change scores after 0.7FO or 1.8FO were compared with the change scores after control oil for each outcome. A greater change score SD after either 0.7FO or 1.8FO compared with control oil was indicative of response heterogeneity (24). However, if the control oil change score SD was larger than either of the FO change score SDs, no further supervised analysis was undertaken, as differences between participants after FO could be explained by random variability alone (24).

### Data analysis strategy

#### Clustering of pre-intervention data

**FIGURE 1:**
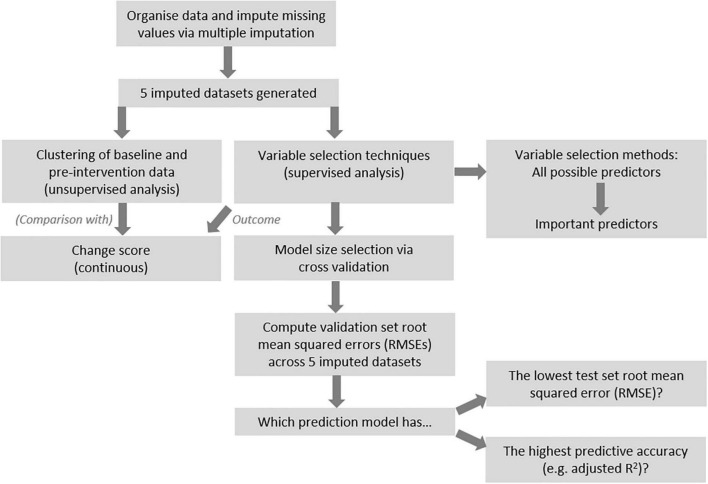
Overview of analysis pipeline.

Figure 1 provides an overview of the procedures for data analysis. After imputation, unsupervised cluster analysis was conducted with all non-outcome variables, in the 0.7FO and 1.8FO datasets, respectively. For each imputed dataset, a dissimilarity matrix was constructed using the “daisy” command within the R *cluster* package. Each value in the matrix referred to the distance between participants, with higher values corresponding to greater dissimilarity (25).

Two different clustering methods were conducted, in order to determine which method led to clearest cluster segregation upon visual inspection. These methods were PAM (Partitioning Around Medoids) also known as k-medoids clustering, where k, the number of clusters, must be stipulated (26); and hierarchical clustering (27), calculating the distance between participants and merging them via application of linkage methods (28). The highest average silhouette value was used to determine the optimal number of clusters after PAM clustering, while the cluster dendrogram informed the number of clusters after hierarchical clustering, with clusters separated using the cutree function. The optimal linkage method for computing the cluster dendrograms was selected by comparing the agglomerative coefficient of four methods (average, single and complete linkage, and Ward’s minimum variance), with the highest value determining the method chosen. These procedures were performed using the *cluster* and *stats* R packages. Final cluster membership was defined as the cluster most frequently assigned to each participant across the 0.7FO and 1.8FO imputed datasets, respectively (≥3/5 of the imputed datasets).

Dimension reduction, via principal components analysis (PCA), was undertaken using the *stats* R package, with results visualized using the *ggbiplot* package. The variables with the greatest loadings on each component were examined.

#### Supervised analysis methods

Several variable selection techniques were chosen to generate models with relevant predictors for each outcome of interest. Results across the 5 imputed datasets were aggregated to form final models and to compare methods. Figure 1 presents a general overview of the analysis procedure.

Using the *leaps* package in R, forward stepwise selection was used to add predictors sequentially that maximally improved the fit of the model to the given outcome. Then, backwards selection was used, starting with a model containing all predictors and sequentially removing predictors that added least to the fit. Both methods were appropriate for the FINGEN dataset since the number of participants was greater than the number of predictors (29).

Next, the shrinkage method LASSO (Least Angle Selection and Shrinkage Operator) was applied using the *glmnet* package in R (30). Briefly, the method applies a parameter, lambda (λ), which shrinks the model coefficients to zero as it increases. Non-zero coefficients therefore represent the most useful predictors. These can be any combination of variables, unlike stepwise selection where predictors are added or subtracted iteratively (29). Finally, a variable selection technique that makes use of a non-linear method, Random Forest regression, was applied—the Boruta algorithm, using the *Boruta* package in R. The algorithm works by comparing the importance of each variable in the dataset to a set of randomly shuffled values, known as shadow features. Variables are confirmed as important or rejected after a series of iterations (31).

#### Model selection and method comparison

For each analysis method, and for each imputed dataset, 10-fold cross-validation or separate training and validation sets were used to select and validate models. For the stepwise selection techniques, 10-fold cross-validation was used to identify the optimal model size that led to the lowest validation set root mean squared error (RMSE)—the amount of error using the remainder of the data not used in model development. Participants were split into 10 random folds using the set seed function in R. For each possible model size (from 1:n, constrained by the number of participants per fold), 9 folds were used as the training set, while 1 fold was used as a test of the model, providing the validation RMSE. This was repeated for each fold, with the average validation RMSE taken across all folds for each model size. To maximize power, the selected model size was run using all data to identify the relevant predictors. For example, if a model containing 3 predictors had the lowest validation RMSE after 10-fold cross-validation, the 3-variable model using the full dataset was examined to identify the resulting variables and coefficients.

The *glmnet* package for LASSO automatically performs 10-fold cross-validation and provides a range of plausible λ values. To determine the optimal λ value and resulting model, validation was performed using a random 2/3 of the data as the training set with the other 1/3 as the validation set. The λ value associated with the lowest validation set RMSE was used to select the corresponding full model. Similarly, for the Boruta algorithm, a random 2/3 of the data was retained in the training set, to maximize shuffling of the shadow features and to improve variable selection. Random Forest regression using the selected variables only was then run with the training data, and used to predict the outcome using the test data, with RMSE calculated.

For stepwise methods, a variable was included in a final pooled linear model if it was included in at least 3 out of 5 of the imputed dataset models. The pooled regression was conducted on all imputed datasets simultaneously using the “with” function in R and *pool* function within the *mice* package (20). Non-zero coefficients that remained across ≥ 3/5 of the LASSO models were averaged and retained as important predictors. Variables identified as important across ≥ 3/5 Boruta models were considered the most relevant for the given outcome.

The method that led to models with the lowest average validation set RMSE across the 5 imputed datasets was considered the best fit for a given outcome, i.e., the model gave the best predictions for change in plasma TG or plasma PC EPA + DHA after intervention. Final models, with the lowest validation set RMSE, are presented in two forms: with numeric coefficients mean-centered but unstandardized, for model interpretability; and with standardized numeric coefficients, for the relative importance of predictors to be compared. For stepwise selection methods, the adjusted R^2^ value quantified the goodness of fit of the models.

Due to anticipated high correlation between change score and pre-intervention value (e.g., TG change vs. pre-intervention TG levels), Oldham’s transformation was performed to determine whether the relationship could be explained by regression to the mean (32). The transformation compares the mean of baseline and final values of an outcome against the change score. If the relationship between change score and pre-intervention value was due to regression to the mean, no significant relationship would remain after the transformation.

## Results

### Outcome change scores

Table 1 shows the average changes in plasma TG and PC EPA + DHA after each intervention arm of the study. A repeated measures ANOVA with Huynh-Feldt correction showed that mean plasma TG change differed significantly between intervention arms [*F*(1.936, 598.2) = 10.19, *p* < 0.001], as has been previously reported (15). Pairwise comparisons using Bonferroni correction revealed that there was a significant reduction in TG concentrations after 0.7FO and 1.8FO compared with control oil, but the difference in TG change between 0.7FO and 1.8FO was not significant (Table 2). For plasma TG change, the change score SD was greater after 1.8FO than after the control oil, but was greater after control oil compared with 0.7FO. This meant that subsequent supervised analyses of TG change after 1.8FO only could be conducted.

**TABLE 1:** Mean change (SD) in plasma TG and plasma PC EPA + DHA in response to fish oil supplementation.

Outcome	Treatment arm	Mean change (SD)
Change in plasma TG (mmol/l) between start and end of 8-week intervention	0.7 g/day EPA + DHA	−0.083 (0.428)
	1.8 g/day EPA + DHA	−0.152 (0.499)
	Control oil	0.011 (0.460)
Change in plasma PC EPA + DHA (% of total fatty acids) between start and end of 8-week intervention	0.7 g/day EPA + DHA	3.05 (1.70)
	1.8 g/day EPA + DHA	4.65 (2.28)
	Control oil	−0.089 (1.40)

**TABLE 2:** Bonferroni-adjusted pairwise comparisons after repeated measures ANOVA for differences in plasma TG change and plasma PC EPA + DHA change between intervention groups.

Outcome/test	Mean difference	Test statistic	Bonferroni-adjusted *p*-value
**Change in plasma TG between start and end of 8-week intervention (mmol/L)**			
0.7 g/day EPA + DHA – control oil	−0.095	−2.594	0.0298
1.8 g/day EPA + DHA – control oil	−0.163	−4.162	0.0001
1.8 g/day EPA + DHA – 0.7 g/day EPA + DHA	−0.069	−2.082	0.1144
**Change in plasma PC EPA + DHA between start and end of 8-week intervention (% of total fatty acids)**			
0.7 g/day EPA + DHA – control oil	3.139	25.44	<0.0001
1.8 g/day EPA + DHA – control oil	4.740	31.45	<0.0001
1.8 g/day EPA + DHA – 0.7 g/day EPA + DHA	1.601	12.32	<0.0001

Repeated measures ANOVA with Huynh-Feldt correction showed that mean PC EPA + DHA change differed significantly between intervention arms [*F*(1.895, 585.5) = 636.1, *p* < 0.001]. Pairwise comparisons with Bonferroni correction revealed that there were significant differences in PC EPA + DHA change between all intervention arms (Table 2), with mean plasma PC EPA + DHA as a proportion of total fatty acids increasing by 3.05 and 4.65% after 0.7FO and 1.8FO, respectively (Table 1). The change score SD was greater after both 0.7FO and 1.8FO compared with control oil, meaning subsequent supervised analyses could be conducted after both fish oil interventions (Table 1).

### Clustering analysis

#### 0.7FO dataset

**FIGURE 2:**
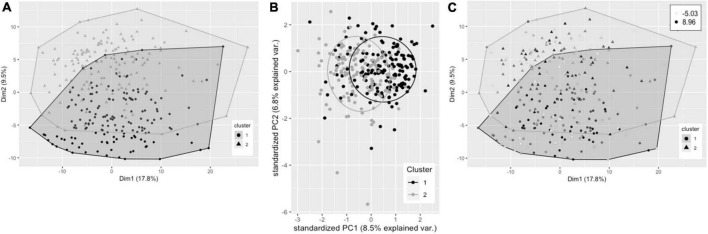
Cluster plots of datasets containing baseline variables and data collected prior to intervention with 0.7 g/day EPA + DHA. Each participant is displayed as one data point, by visualizing the clusters using the first of the imputed datasets. **(A)** Visualization of hierarchical clusters, cluster 1 _°_ (black, *n* = 161), cluster 2 Δ (gray, *n* = 149). **(B)** PCA plot of pre-0.7 g/day data visualizing clusters across the first two principal components (clusters as described in **A**). **(C)** Clustering as shown in a with gradation of shading relating to change in plasma PC EPA + DHA (as% of total fatty acids) after 0.7 g/day EPA + DHA intervention, with darker shading corresponding to greatest increases in EPA + DHA. Legend in top right shows range of EPA + DHA change. PC, plasma phosphatidylcholine; PCA, principal components analysis.

Hierarchical clustering using Ward’s method led to clearest discrimination of clusters, resulting in two clusters with 161 and 149 participants in clusters 1 and 2, respectively (Figure 2A). PCA revealed a degree of separation of the two clusters across the first two principal components (PCs) (Figure 2B). There was no significant difference in plasma TG change after 0.7FO between the two clusters. Mean change in plasma PC EPA + DHA for participants in cluster 1 (3.22%) was not significantly greater than EPA + DHA change for participants in cluster 2 (2.86%), *p* = 0.058 (Figure 2C).

#### 1.8FO dataset

**FIGURE 3:**
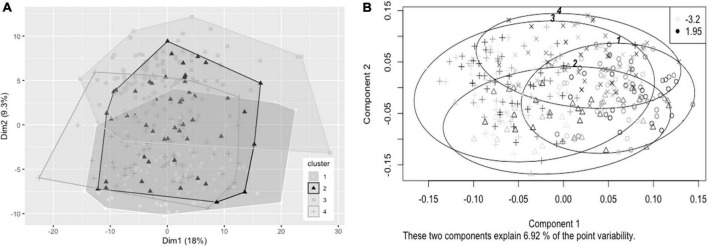
Cluster plots of datasets containing baseline variables and data collected prior to intervention with 1.8 g/day EPA + DHA. Each participant is displayed as one data point. **(A)** Visualization of hierarchical clusters using the first imputed dataset, cluster 1 _°_ (white, *n* = 82), cluster 2 Δ (black, *n* = 51), cluster 3 △ (light gray, *n* = 112); cluster 4 + (dark gray, *n* = 65). **(B)** Visualization of hierarchical clusters using the fourth imputed dataset, with gradation of shading relating to change in plasma TG concentration (mmol/L) after 1.8 g/day EPA + DHA intervention, with lightest shading corresponding to greatest reductions in plasma TG concentration. Legend in top right shows range of plasma TG change. TG, triglyceride.

Hierarchical clustering using Ward’s method was also found to lead to the clearest discrimination of clusters with the 1.8FO dataset, with four clusters found to be optimal (1, *n* = 82; 2, *n* = 51; 3, *n* = 112; 4, *n* = 65) (Figure 3A). Clusters did not segregate clearly upon application of PCA. Due to differences in imputed values between datasets for plasma TG change, a significant difference in TG change between clusters was observed in one of the imputed datasets only [*F*(3, 206) = 2.67, *p* < 0.05], with participants in cluster 3 having a mean reduction in plasma TG of −0.247 mmol/L, significantly greater than a mean reduction of −0.052 mmol/L for participants in cluster 1 (*p* < 0.05, Bonferroni corrected) (Figure 3B). The difference in EPA + DHA change between clusters was not significantly different (*p* = 0.073).

### Supervised analysis

#### Predicting plasma triglycerides change after 1.8FO

**FIGURE 4:**
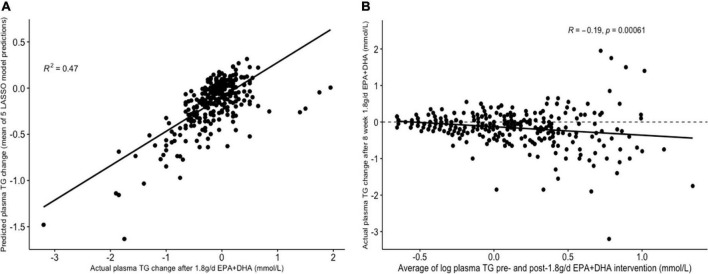
Graphs depicting results from supervised analysis with plasma TG change after 1.8 g/day EPA + DHA as intervention. **(A)** Scatter plot comparing actual TG change against predicted TG change using the LASSO model, averaged across all imputed datasets; **(B)** scatter plot depicting the correlation between the average of logged plasma TG values pre- and post-1.8g/day EPA + DHA intervention with observed TG change. Dashed line represents no change. LASSO, Least Angle Selection and Shrinkage Operator; TG, triglyceride.

Table 3 presents the average RMSEs from supervised analysis of the five imputed datasets. For predicting plasma TG change, the lowest average RMSE across all five imputed datasets corresponded to models generated by LASSO. Table 4 presents the mean-centered and standardized shrunk coefficients, averaged across all imputed datasets. In total, 18 predictors were selected across 3 or more imputed datasets. The highest positive coefficient corresponded to baseline plasma insulin concentration, while the highest negative coefficient corresponded to pre-intervention TG concentration. These two variables were also selected by the other supervised analysis methods. For the other numeric predictors, the standardized coefficients were all less than ± 0.1, with the next largest coefficients corresponding to baseline LDL and the fatty acid C20:3*n*-6, both positively associated with TG change; and baseline IL-10 levels, negatively associated with TG change. For the categorical variables, carriers of the T allele for *rs1800588*, a polymorphism of the LIPC gene, was also positively associated with TG change. Figure 4A shows the relationship between predicted plasma TG change using the LASSO model, and actual plasma TG change, with an R^2^ upon application to the original (un-imputed) dataset of 0.47. Upon applying Oldham’s transformation, Figure 4B shows a significant negative correlation (*R* = −0.19, *p* < 0.001) between the average of (log-transformed) pre- and post-intervention TG values against observed TG change, indicating that participants with higher pre-intervention plasma TG show greater reduction after 1.8FO, after adjusting for regression to the mean.

**TABLE 3:** Model RMSEs after application of supervised analysis methods to the outcomes plasma TG change after 1.8 g/day EPA + DHA, plasma PC EPA + DHA change after 0.7 g/day EPA + DHA, and plasma PC EPA + DHA change after 1.8 g/day EPA + DHA.

Outcome	Plasma TG change after 1.8 g/day EPA + DHA	Plasma PC EPA + DHA change after 0.7 g/day EPA + DHA	Plasma PC EPA + DHA change after 1.8 g/day EPA + DHA

Method	Mean RMSE (SD), 5 imputed datasets		
Forward stepwise	0.396 (0.006)	**1.470** (0.024)	1.982 (0.032)
Backward stepwise	0.400 (0.010)	1.488 (0.015)	**1.966** (0.013)
LASSO	**0.353** (0.058)	1.521 (0.051)	2.059 (0.170)
Boruta—test set RMSE	0.452 (0.064)	1.610 (0.127)	2.177 (0.106)

Lowest RMSEs for each outcome are given in bold.

**TABLE 4:** Shrunk coefficients after LASSO analysis for predicting plasma TG change after 1.8 g/day EPA + DHA.

Variable name	Mean-centered coefficient (SD)	Standardized coefficient
Intercept	−0.330 (0.103)	0
APOE—APOE4 variant	−0.010 (0.006)	
Baseline BMI (kg/m^2^)	0.002 (0.001)	0.017
Baseline CRP (mg/l)	0.005 (0.002)	0.030
Baseline plasma insulin (mmol/L)	0.014 (0.003)	0.118
Baseline IL-10 (pg/ml)	−0.007 (0.002)	−0.045
Baseline VCAM-1 (ng/ml)	<−0.001	−0.030
Pre-intervention plasma TG (mmol/L)	−0.442 (0.048)	−0.577
Pre-intervention LDL-cholesterol (mmol/L)	0.035 (0.006)	0.066
Fruit consumption (g)	<0.001	−0.011
Saturated fat consumption (g)	0.001 (0.001)	0.040
*rs320* (G > T)	−0.015 (0.004)	
*rs2250656* (C > T)	−0.017 (0.009)	
*rs1800588* (T > C)	0.058 (0.031)	
*rs1800795* (C > G)	0.024 (0.012)	
*rs1800896* (C > T)	0.015 (0.009)	
*rs5370* (T > G)	0.054 (0.030)	
C20:3*n*-6	0.027 (0.012)	0.049
C20:4*n*-6	0.006 (0.002)	0.024

Variables listed were selected by 3 or more of the 5 imputed datasets, and depict the mean (SD) of their shrunk coefficients across all imputed datasets for which they were selected. Both mean-centered (left) and standardized (right, variables on continuous numeric scale only) shrunk coefficients are presented. APOE/APOE4, apolipoprotein E3/E4 or E4/E4; CRP, C-reactive protein; IL-10, interleukin 10; LDL, low-density lipoprotein; TG, triglyceride; VCAM-1, vascular cell adhesion protein 1.

#### Predicting plasma PC EPA + DHA change after 0.7FO

**FIGURE 5:**
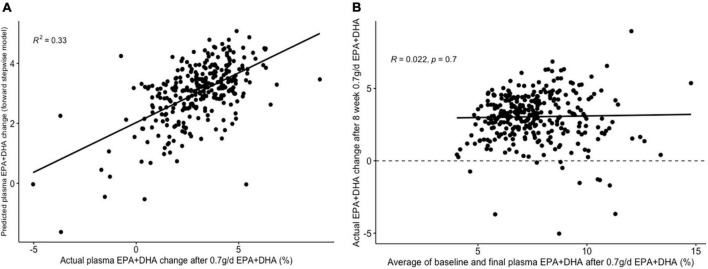
Graphs depicting results from supervised analysis with plasma PC EPA + DHA change after 0.7 g/day EPA + DHA intervention. **(A)** Scatter plot comparing actual PC EPA + DHA change against predicted change using the final forward stepwise model. **(B)** Scatter plot depicting the correlation between the average of pre- and post-intervention plasma PC EPA + DHA proportion against observed change in EPA + DHA proportions. Dashed line represents no change. PC, plasma phosphatidylcholine.

The lowest average RMSE for predicting plasma PC EPA + DHA change after 0.7FO corresponded to models generated by forward stepwise selection (Table 3). Table 5 shows both the mean-centered coefficients, pooled from the 5 imputed datasets, and standardized coefficients calculated from running the model against the standardized non-imputed dataset, with an adjusted R^2^ value of 0.32. The final model contained 6 predictors with positive coefficients for age, sex, a SNP in the tumor necrosis factor alpha (TNFα) gene (*rs1800629*) and pre-intervention PC docosapentaenoic acid (DPA) proportion, and negative coefficients for pre-intervention proportions of EPA and DHA. Figure 5A shows the relationship between predicted and actual EPA + DHA change using the forward stepwise model, with an R^2^ of 0.33 after application to the un-imputed dataset. After application of Oldham’s transformation, Figure 5B shows no relationship between the average of pre- and post-intervention EPA + DHA with observed EPA + DHA change, indicating that the relationship between pre-intervention EPA + DHA and subsequent EPA + DHA change after 0.7FO can be explained by regression to the mean.

**TABLE 5:** Model output after performing forward stepwise regression for predicting plasma PC EPA + DHA change after 0.7 g/day EPA + DHA.

Pooled mean centered regression coefficients					Standardized regression coefficients, un-imputed dataset				
Term	Estimate	Std. error	Test statistic	*p*	Term	Estimate	Std. error	Test statistic	*p*
Intercept	2.536	0.129	19.61	<0.001	Intercept	2.686	0.119	22.49	<0.001
Age	0.021	0.006	3.280	0.001	Age	0.281	0.085	3.300	0.001
Sex—Female	0.681	0.165	4.139	<0.001	Sex–Female	0.694	0.170	4.094	<0.001
*rs1800629* – G/A	0.400	0.178	2.243	0.026	*rs1800629* (G > A)	0.230	0.082	2.806	0.005
*rs1800629*—A/A	0.649	0.337	1.926	0.055					
C20:5*n*-3	−0.859	0.118	−7.285	<0.001	C20:5*n*-3	−0.727	0.102	−7.119	<0.001
C22:5*n*-3	1.514	0.346	4.371	<0.001	C20:5*n*-3	0.376	0.091	4.124	<0.001
C22:6*n*-3	−0.247	0.077	−3.206	0.001	C20:5*n*-3	−0.325	0.101	−3.218	0.001

Data showing mean-centered regression coefficients pooled across all imputed datasets (left), and upon applying the model to the standardized un-imputed dataset (right, continuous numeric scale variables standardized only).

#### Predicting plasma PC EPA + DHA change after 1.8FO

**FIGURE 6:**
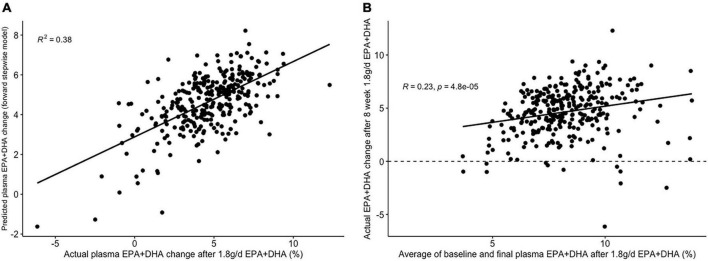
Graphs depicting results from supervised analysis with plasma PC EPA + DHA change after 1.8 g/day EPA + DHA intervention. **(A)** Scatter plot comparing actual PC EPA + DHA change against predicted change using the final backward stepwise model; **(B)** scatter plot depicting the correlation between the average of pre- and post-intervention plasma PC EPA + DHA proportion against observed change in EPA + DHA proportions. Dashed line represents no change. PC, plasma phosphatidylcholine.

The lowest average RMSE for predicting plasma PC EPA + DHA change after 1.8FO corresponded to models generated by backward stepwise selection (Table 3). The final model contained 11 predictors with positive coefficients for age, sex and a SNP in the Fatty Acid Binding Protein 1 (FABP1) gene (*rs2241883*), and negative coefficients for body mass index (BMI) and a number of pre-intervention PC fatty acids, as shown in Table 6. Figure 6A shows the relationship between predicted and actual EPA + DHA change using the backward stepwise model, with an R^2^ of 0.38 after application to the un-imputed dataset. After application of Oldham’s transformation, Figure 6B shows a significant positive correlation (*R* = 0.23, *p* < 0.001) between the average of pre- and post-intervention PC EPA + DHA and observed PC EPA + DHA change, meaning that after accounting for regression to the mean, there was a greater change in PC EPA + DHA for participants with higher pre- and post-intervention average PC EPA + DHA proportions.

**TABLE 6:** Model output after performing backward stepwise regression for predicting plasma PC EPA + DHA change after 1.8 g/day EPA + DHA.

Pooled mean centered regression coefficients					Standardized regression coefficients, un-imputed dataset				
Term	Estimate	Std. error	Test statistic	*p*	Term	Estimate	Std. error	Test statistic	*p*
Intercept	3.915	0.193	20.27	0	Intercept	4.235	0.157	26.95	<0.001
Age	0.043	0.009	4.777	<0.001	Age	0.563	0.115	4.897	<0.001
Sex—Female	0.799	0.224	3.572	<0.001	Sex—Female	0.774	0.224	3.451	0.001
BMI	−0.088	0.035	−2.537	0.012	BMI	−0.320	0.118	−2.716	0.007
*rs2241883*—T/C	0.564	0.229	2.462	0.014	*rs2241883* (T > C)	0.323	0.107	3.012	0.003
*rs2241883*—C/C	0.806	0.343	2.350	0.019					
C16:0	−0.429	0.109	−3.922	<0.001	C16:0	−0.844	0.219	−3.852	<0.001
C18:0	−0.281	0.109	−2.572	0.011	C18:0	−0.496	0.197	−2.515	0.012
C18:1*n*-7	−0.350	0.120	−2.904	0.004	C18:1*n*-7	−0.488	0.173	−2.813	0.005
C18:2*n*-6	−0.454	0.091	−5.009	<0.001	C18:2*n*-6	−1.304	0.263	−4.966	<0.001
C20:4*n*-6	−0.491	0.111	−4.408	<0.001	C20:4*n*-6	−0.903	0.211	−4.287	<0.001
C20:5*n*-3	−1.670	0.202	−8.275	<0.001	C20:5*n*-3	−1.337	0.163	−8.217	<0.001
C22:6*n*-3	−0.548	0.112	−4.882	<0.001	C22:6*n*-3	−0.702	0.142	−4.935	<0.001

Data showing mean-centered regression coefficients pooled across all imputed datasets (left), and upon applying the model to the standardized un-imputed dataset (right, continuous numeric scale variables standardized only).

To examine the different results after Oldham’s transformation with 0.7FO and 1.8FO more closely, the relationship between pre- and post-intervention PC EPA + DHA with PC EPA + DHA change were examined separately (Supplementary Figure 2). For both fish oil doses, there was a negative association between pre-intervention plasma PC EPA + DHA and subsequent PC EPA + DHA change, of a similar magnitude for both fish oil doses (Supplementary Figures 1A,B). However, when comparing post-intervention PC EPA + DHA proportion with PC EPA + DHA change, there was a higher positive correlation after 1.8FO (*R* = 0.68, Supplementary Figure 1D) than after 0.7FO (*R* = 0.46, Supplementary Figure 1C), with PC EPA + DHA increase more uniform after 1.8FO than after 0.7FO.

## Discussion

Nutrition studies typically reveal substantial heterogeneity in physiological response after an intervention. Studies that collect data on a large array of predictors of response, in a sufficient number of participants, can be utilized to identify potential predictors of this response variability. This is of interest in the growing fields of precision and personalized nutrition, where elucidation of predictors of response may help to identify the characteristics of people most and least likely to respond beneficially. The results of this analysis revealed that the application of variable selection techniques, in particular, can identify new and clinically important predictors that explain between a third to a half of the variability in change in plasma TG and PC EPA + DHA, after an intervention with fish oil. Our predictive models showed greater TG-lowering with lower pre-intervention levels of plasma insulin, LDL cholesterol and C20:3*n*-6 levels, along with C carriers (compared with T carriers) of the SNP *rs1800588*; and greater TG-lowering in those with higher pre-intervention levels of plasma TG (additional to regression to the mean) and serum IL-10. For predicting change in plasma PC EPA + DHA, greater increases were observed with higher age and female sex, along with lower levels of baseline plasma C20:5*n*-3 (EPA) and C22:6*n*-3 (DHA), for both doses of fish oil. However, the relationship between baseline EPA + DHA levels and degree of change differed between the 0.7FO and 1.8FO fish oil interventions, with the relationship for 0.7FO explained by regression to the mean, while increases in EPA + DHA after 1.8FO were more uniform. This means that greater increases in EPA + DHA than expected were observed in those with higher baseline EPA + DHA levels.

Change in plasma TG and plasma PC EPA + DHA were the outcomes of interest in this study and were used on a continuous scale rather than being dichotomized into “responders” or “non-responders” to the intervention to maximize statistical power (33, 34). Findings from this study identify important physiological predictors of response heterogeneity at a group level for the given outcomes of interest. The final models were generated through application of different variable selection methods—with forward and backward stepwise selection, and LASSO, generating the models with the lowest RMSE for predicting change in plasma TG after 1.8FO and in PC EPA + DHA after 0.7FO and 1.8FO. Stepwise selection methods such as forward and backward stepwise selection have been criticized (35, 36) as they are often overfit to training data and undergo lack of validation, or are used as the sole model-building approach. In this study, we mitigated these limitations by using cross-validation to select the final model size, repeating the process across 5 imputed datasets to determine the most appropriate predictors to retain in the final model, and comparing the validation set RMSEs with models generated by other variable selection methods. While cross-validation helps to prevent model overfitting, it will be important to validate these models using external, independent datasets to ascertain whether findings from the FINGEN study are generalizable to other populations (37).

The variables selected by LASSO for predicting plasma TG change after 1.8FO (Table 4) included baseline BMI, plasma insulin concentration and saturated fat intake, and pre-intervention LDL-cholesterol concentration, all of which had positive (shrunk) coefficients, meaning that higher values of these predictors were associated with less TG-lowering. Each of these predictors is known to be associated with higher TG concentrations, with obesity and insulin resistance being features of the metabolic syndrome (38). Conversely, other predictors had negative coefficients, including APOE4 carriers, meaning this variant was associated with greater plasma TG-lowering than other APOE genotypes. This supports the previous findings from the FINGEN cohort for a non-significant trend in greater TG reductions in APOE4 carriers, with the greatest TG reductions in men carrying APOE4 (15). Baseline concentration of plasma interleukin 10 (IL-10) and self-reported fruit consumption were also among the predictors with negative coefficients; higher values of both are associated with better health status, and these participants were more likely to show falls in plasma TG in response to the intervention. Apart from the association of higher pre-intervention plasma TG concentration with greater TG-lowering, the variables selected by LASSO suggest that participants with a profile indicative of lower heart disease risk are more likely to have greater plasma TG-lowering after 1.8 g/day EPA + DHA.

Participants who were older and female tended to have the greatest increases in plasma PC EPA + DHA (Tables 5, 6), confirming findings from a previous study (39). For change after 1.8FO only, higher BMI was associated with a lower increase in PC EPA + DHA, in line with previous findings (39). For predicting PC EPA + DHA change after 1.8FO, higher pre-intervention levels of the saturated fatty acids palmitic (C16:0) and stearic acid (C18:0), the trans fatty acid vaccenic acid (C18:1*n*-7) and the unsaturated fatty acids linoleic acid (C18:2*n*-6) and arachidonic acid (C20:4*n*-6) were associated with a lesser increase in PC EPA + DHA (Table 6), which has, to the best of our knowledge, not been reported before. On the other hand, for the model predicting PC EPA + DHA change after 0.7FO, a higher proportion of DPA in plasma PC was associated with greater increases in PC EPA + DHA in response to supplementation. As desaturation of DPA leads to the formation of DHA, DHA levels are likely to increase if more DPA is available (40), and DPA supplementation has been shown to increase DHA levels in plasma TG (41). As plasma PC fatty acid proportions were included in this analysis, this suggests that lower levels of other fatty acids will enable EPA + DHA to form a greater proportion of total plasma PC fatty acids. Unsurprisingly, higher pre-intervention concentrations of EPA (C20:5*n*-3) and DHA (C22:6*n*-3) were associated with a smaller increase in PC EPA + DHA after both fish oil interventions, as has been observed previously (39). The standardized coefficients for pre-intervention EPA were approximately twice as large as the coefficients for DHA (Tables 5, 6), suggesting that EPA status was a more important predictor of incorporation of EPA + DHA into PC. This makes sense given that DHA is a downstream metabolite of EPA (40). Interestingly, different results were observed upon applying Oldham’s transformation to EPA + DHA change after each fish oil intervention. As the relationship between the average of pre- and post-intervention EPA + DHA with EPA + DHA change was not significant for 0.7FO, this suggests the relationship can be explained by regression to the mean. However, the significant positive association that remained after 1.8FO suggests that a greater increase in EPA + DHA occurred than would be expected in those with higher pre-intervention EPA + DHA. This finding supports a lack of a “ceiling effect,” meaning higher pre-intervention plasma PC EPA + DHA levels do not limit further increases in EPA + DHA in response to supplementation. The findings of the JELIS trial lend support to this claim, where Japanese participants had a reduction in coronary events after EPA supplementation, despite high habitual consumption of fish and thus high pre-intervention plasma LC n-3 PUFA status (42).

A strength of this analysis approach was the use of a large dataset with many variables, with the potential to uncover new variables associated with change in plasma TG and PC EPA + DHA levels. Furthermore, the crossover design enabled analyses to be performed on the same participants, enabling better comparisons to be made between the results for EPA + DHA change after both 0.7FO and 1.8FO. However, the analysis may have been limited by the statistical power of the dataset, with a large number of predictors considered in relation to the number of participants. Despite this, the supervised analysis methods applied in this paper were suitable for use on high-dimensional datasets, where the power is even smaller due to the number of predictors being greater than the number of participants (27). These types of dataset are increasingly common in an era of precision medicine, where information on an array of markers including genotype, metabolomics and microbiome are increasingly collected (1, 43). While limiting the number of variables considered in this analysis would have improved statistical power, this would not have made full use of the dataset, nor enabled potential discovery of new predictors of response to the outcomes of interest. Using validation approaches such as cross-validation to determine the size of models selected, and performing analyses across 5 imputed datasets, also increased the likelihood that models contained relevant variables, as final models considered variables that were only in common across at least 3 of the 5 imputed datasets.

In conclusion, the application of supervised analysis approaches, particularly variable selection methods, led to the identification of new variables for predicting change in plasma TG and plasma PC EPA + DHA after fish oil supplementation. This means that females and those who are older are more likely to benefit from fish oil supplements in terms of increasing the omega-3 index. In addition, those with higher levels of plasma TG and certain inflammatory markers, together with lower levels of plasma insulin, LDL cholesterol, C20:3*n*-6, and saturated fat consumption, are more likely to benefit from fish oil supplements in terms of TG lowering, based on the results of this study. A similar analysis approach applied to data from other large fish oil supplementation studies could provide an external validation of our models, or help to identify additional markers of response. Our study highlights how application of appropriate statistical methods to rich datasets can develop our knowledge of the factors underpinning physiological response heterogeneity to interventions, and hence provide a useful tool for precision nutrition and in the future tailoring of dietary recommendations.

## Data availability statement

Analytic code can be made available upon request to the corresponding author pending application and approval. Requests to access the datasets should be directed to corresponding author.

## Ethics statement

The studies involving human participants were reviewed and approved by the research ethics committees at each of the four universities involved in the original study (https://doi.org/10.1093/ajcn/88.3.618). The participants provided their written informed consent to participate in this study.

## Author contributions

TP, AW, EZ, PZ, and BdR conceptualized and designed the research. TP conducted the research, analyzed the data, and wrote the manuscript. AM, PC, and JM designed and conducted the original FINGEN study and HF carried out the fatty acid analysis. All authors reviewed and approved the final manuscript.

## Abbreviations

0.7FO: 0.7 g/day EPA + DHA from fish oil
1.8FO: 1.8 g/day EPA + DHA from fish oil
APOE(4): apolipoprotein E(4)
DPA: docosapentaenoic acid
FABP1: Fatty Acid Binding Protein 1
FO: fish oil
LASSO: Least Angle Selection and Shrinkage Operator
LC n-3 PUFAs: long-chain n-3 polyunsaturated fatty acids
PC: plasma phosphatidylcholine
PCA: principal components analysis
RMSE: root mean squared error
TG: triglycerides.
