# Supplementary material for: Models predict change in plasma triglyceride concentrations and long-chain n-3 polyunsaturated fatty acid proportions in healthy participants after fish oil intervention
Source: Front Nutr. 2022 Oct 25;9:989716. doi: 10.3389/fnut.2022.989716 (PMC9641003; doi:10.3389/fnut.2022.989716)
Supplement: Supplementary file 1 [file Data_Sheet_1.pdf]

frontiers

Supplementary Material

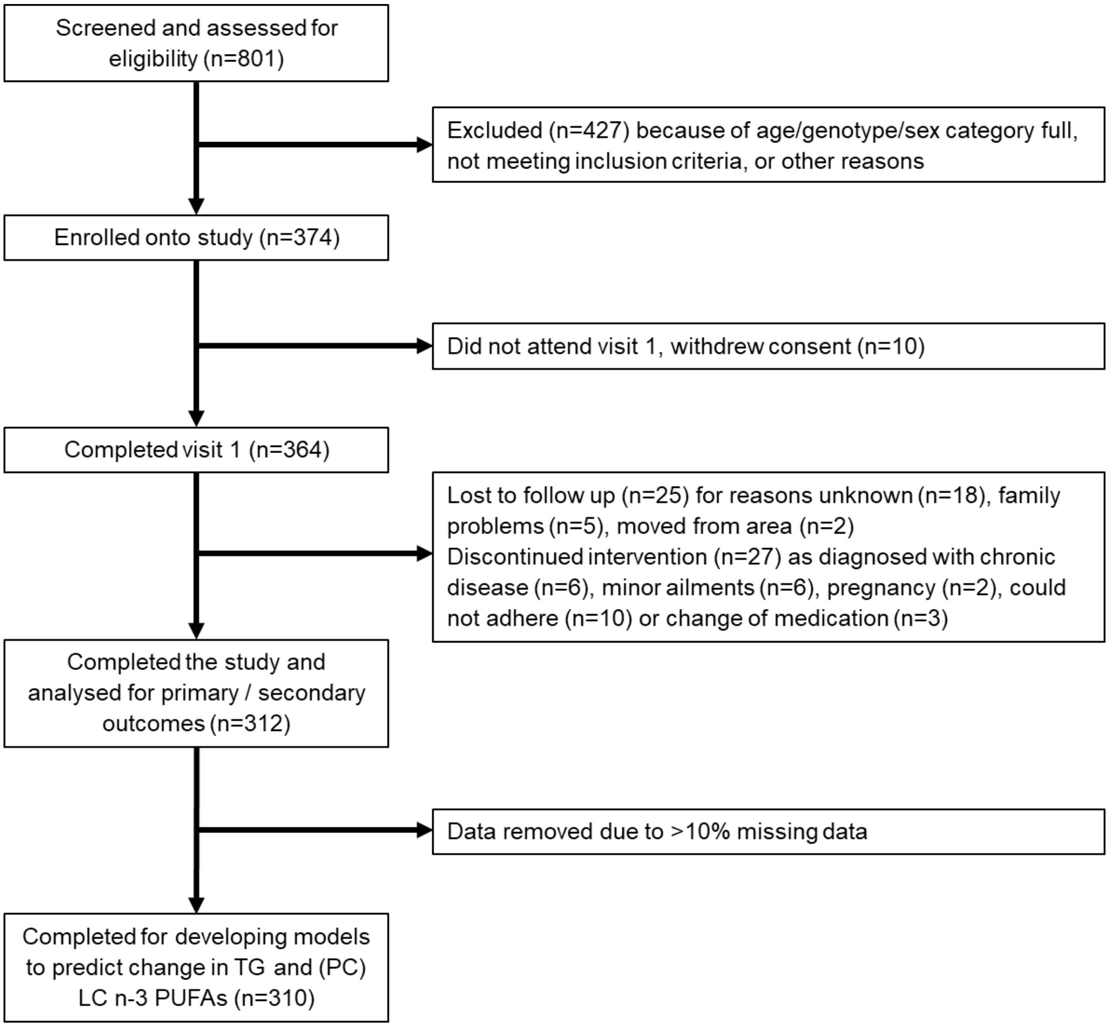

Supplementary Figure 1. Participant flow diagram**.**

Supplementary Table 1 List of all variables included in the dataset

**Variable name/ code**	**Variable description/levels**	**Gene name**	**SNP reference ID(s)**
Vol_no	Volunteer identifier		

Age	Volunteer age (y) at baseline		
Gender	Male (0), Female (1)		
apoE_code	2 = E2/E2 + E2/E3, 3 = E3/E3, 4 = E3/E4 + E4/E4	APOE	*rs429358* and *rs7412*
eNOS_code	1 = GG, 2 = GT, 3 = TT	eNOS	*rs1799983*
BMI	Volunteer Body Mass Index at baseline		
Alcohol	Units of alcohol consumed/wk		
APOA1_v1	Plasma APOA1 levels at baseline		
APOE_v1	Plasma APOE levels at baseline		
CRP_v1	Plasma CRP levels at baseline		
Glu_v1	Plasma glucose levels at baseline		
HDLC_v1	Plasma HDL-cholesterol levels at baseline		
Ins_v1	Plasma insulin levels at baseline		
NEFA_v1	Plasma NEFA levels at baseline		
Psel_v1	Plasma P-selectin levels at baseline		
Esel_v1	Plasma E-selectin levels at baseline		
IL-6_v1	Plasma IL-6 levels at baseline		
IL-10_v1	Plasma IL-10 levels at baseline		
PAI1_v1	Plasma PAI-1 levels at baseline		
tPA_v1	Plasma tPA levels at baseline		
ICAM_v1	Plasma ICAM levels at baseline		
VCAM_v1	Plasma VCAM levels at baseline		

TNF_v1	Plasma TNF levels at baseline		
vWF_v1	Plasma vWF levels at baseline		
DBP_a/c_0	Diastolic blood pressure levels before intervention period		
SBP_a/c_0	Systolic blood pressure levels before intervention period		
TG_a/c_0	Plasma triglyceride levels before intervention period		
TG_a/c_8	Plasma triglyceride levels after intervention period (used to calculate TG change score only)		
TG_a/c_change	Triglyceride change between start and end of intervention period (outcome)		
TC_a/c_0	Total plasma cholesterol levels before intervention period		
LDL_a/c_0	Plasma LDL-cholesterol levels before intervention period		
Fruit_g_d	Fruit consumption in grams at baseline		
Veg_g_d	Vegetable consumption in grams at baseline		
Fat_percent	% of calories from fat at baseline		
Mono_fat	Grams of monosaturated fat at baseline		
Polyunsat_fat	Grams of polyunsaturated fat at baseline		
Sat_fat	Grams of saturated fat at baseline		
Energy_MJ	Energy consumed per day, MJ		
Oilyfish_wk	Portions of oily fish consumed/wk		
apoA5_1131	1 = AA, 2 = GA	APOA5	*rs662799*
apoA5_S19W	1 = GG, 2 = CG, 3 = CC		*rs3135506*

LPL_Hind_III	1 = TT, 2 = TG, 3 = GG	LPL	*rs320*
LPL_S447X	1 = CC, 2 = GC, 3 = GG		*rs328*
apoC3_455	1 = TC, 2 = TT, 3 = CC	APOC3	*rs2854116*
apoA4_S347Th	1 = AA, 2 = GA, 3 = GG	APOA4	*rs5675*
Comp_C3_1	1 = GG, 2 = GA, 3 = AA	C3	*rs11569562*
Comp_C3_2	1 = TT, 2 = TC, 3 = CC		*rs2250656*
LIPC_250	1 = GG, 2 = GA, 3 = AA	LIPC	*rs2070895*
LIPC_514	1 = CC, 2 = TC, 3 = TT		*rs1800588*
CYP7A1_204	1 = GG, 2 = TG, 3 = TT	CYP7A1	*rs3808607*
TNFa_238	1 = GG, 2 = AG, 3 = AA	TNF-α	*rs361525*
TNFa_308	1 = GG, 2 = GA, 3 = AA		*rs1800629*
TNFa_308_a/c_0	Measure of TNF-α levels before intervention period		
LTA_252	1 = AA, 2 = GA, 3 = GG	LTA	*rs909253*
IL1B_511	1 = CC, 2 = TC, 3 = TT	IL-1β	*rs16944*
IL1B_6054	1 = GG, 2 = AG, 3 = AA		*rs1143643*
IL6_174	1 = GG, 2 = CG, 3 = CC	IL-6	*rs1800795*
IL10_1082	1 = TT, 2 = CT, 3 = CC	IL-10	*rs1800896*
NFkB_94	1 = ATTG:-, 2 = ATTG:ATTG, 3 = -:-	NFκB	*rs28720239*
LepR_G_A	1 = CC, 2 = TC, 3 = TT	LEPR	*rs3790433*
Adiponectin_276	1 = CC, 2 = CA, 3 = AA	Adiponectin	*rs1501299*

IR_S1	1 = CC, 2 = TC, 3 = TT	IRS1	*rs1801278*
PI3Kinase	1 = GG, 2 = GA, 3 = AA	P13Kinase	*rs3730089*
VLDL_R	1 = AA, 2 = CA, 3 = CC	VLDLR	*rs1454626*
p22phox_242	1 = CC, 2 = CT, 3 = TT	NADPHox	*rs4673*
p22phox_nitrate_a/c_0	Measure of NADPHox before intervention period		
EDN1_198	1 = GG, 2 = TG, 3 = TT	EDN1	*rs5370*
UTS2_T21M	1 = AA, 2 = GA, 3 = GG	UTS2	*rs228648*
UTS2R_8515	1 = CC, 2 = TC, 3 = TT	UTS2R	*rs35373706*
CYP1A1_2_I462V	1 = AA, 2 = GA, 3 = GG	CYP1A1.2	*rs1048943*
FADS174575	1 = CC, 2 = CG, 3 = GG	FADS2	*rs174575*
FADS174575_ADMA_a/c_0	Measure of FADS2 before intervention period		
ELOVL1	1 = GG, 2 = GA, 3 = AA	ELOVL2	*rs953413*
FABP1_Th94Al	1 = TT, 2 = TC, 3 = CC	FABP1	*rs2241883*
PPARa_L162V	1 = CC, 2 = GC, 3 = GG	PPARα	*rs1800206*
PPARa_3UTR	1 = GG, 2 = GA, 3 = AA		*rs6008259*
PPARa_3UTR_RQ_a_0	Measure of PPARα before intervention period		
PPARa_5UTR	1 = CC, 2 = CA, 3 = AA		*rs535543*
SCARB1	1 = CC, 2 = TC, 3 = TT	SCARB1	*rs4238001*
FABP2	1 = TT, 2 = AT, 3 = AA	FABP2	*rs1799883*
CD36_1	1 = GG, 2 = AG, 3 = AA	CD36	*rs1527483*
CD36_2	1 = GG, 2 = GC, 3 = CC		*rs1049673*

CD36_3	1 = AA, 2 = GA, 3 = GG		*rs1761667*
CD36_4	1 = AA, 2 = GA, 3 = GG		*rs1984112*
C14_0_a/c_0	Plasma fatty acid levels of C14:0 before intervention period		
C16_0_a/c_0	Plasma fatty acid levels of C16:0 before intervention period		
C18_0_a/c_0	Plasma fatty acid levels of C18:0 before intervention period		
C16_1_a/c_0	Plasma fatty acid levels of C16:1 before intervention period		
C18_1_a/c_0	Plasma fatty acid levels of C18:1 before intervention period		
C18_2_a/c_0	Plasma fatty acid levels of C18:2 before intervention period		
C20_3_a/c_0	Plasma fatty acid levels of C20:3 before intervention period		
C20_4_a/c_0	Plasma fatty acid levels of C20:4 before intervention period		
C20_5_a/c_0	Plasma fatty acid levels of C20:5 before intervention period		
C22_5_a/c_0	Plasma fatty acid levels of C22:5 before intervention period		
C22_6_a/c_0	Plasma fatty acid levels of C22:6 before intervention period		
O3I_a/c_0	Plasma EPA+DHA levels before intervention period (used to calculate TG change score only)		
O3I_a/c_change	Change in plasma EPA+DHA levels between start and end of intervention period (outcome)		

APOA4 – apolipoprotein A4 gene; APOA5 – apolipoprotein A5 gene; APOC3 – apolipoprotein C3 gene; APOE – apolipoprotein E gene; C3 – complement component 3 gene; CD36 – scavenger receptor gene; CYP1A1.2 – cytochrome

p450 gene; CYP7A1 – cholesterol 7 alpha hydrolase gene; EDN1 – Endothelin-1 gene; ELOVL2 – fatty acid elongase gene; eNOS – endothelial nitric oxide synthase gene; FABP1 – fatty acid binding protein 1 gene; FABP2 – fatty acid binding protein 2 gene; FADS2 – delta 6 desaturase gene; IL-6 – interleukin 6 gene; IL-10 – interleukin 10; IL-1β – interleukin 1 beta gene; IRS1 – insulin receptor substrate1 gene; LEPR – leptin receptor gene; LIPC – hepatic lipase gene; LPL – lipoprotein lipase gene; NADPHox – nicotinamide adenine dinucleotide phosphate oxidase; PIK3R1 – P13 Kinase regulatory subunit 1α gene; PPARα - peroxisome proliferator activator receptor alpha; SCARB1 – scavenger receptor class B1 gene; TNF-α - tumour necrosis factor alpha gene; UTS2 – urotensin 2 gene; UTS2R urotensin 2 receptor gene; VLDLR – very low density lipoprotein receptor gene.

Supplementary Figure 2

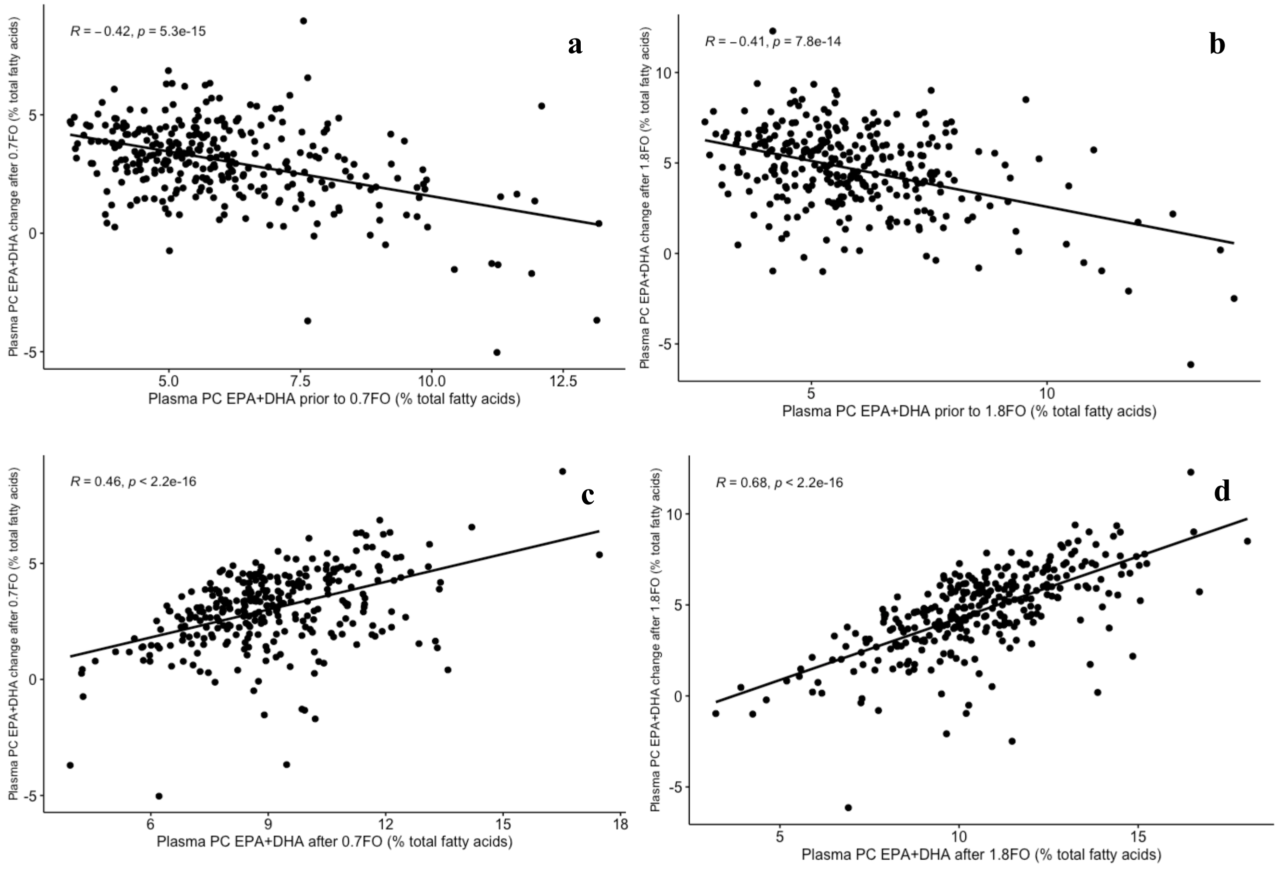

Supplementary Figure 2. Relationship between initial and final plasma PC EPA+DHA with plasma PC EPA+DHA change. **a** relationship between pre-0.7FO plasma PC EPA+DHA with EPA+DHA change after 0.7FO; **b** relationship between pre-1.8FO plasma PC EPA+DHA with EPA+DHA change after 1.8FO; **c** relationship between plasma PC EPA+DHA after 0.7FO with EPA+DHA change; **d** relationship between plasma PC EPA+DHA after 1.8FO with EPA+DHA change. 0.7FO – 0.7 g/d EPA+DHA; 1.8FO – 1.8 g/d EPA+DHA; PC – plasma phosphatidylcholine.
